# Medical Wards of Mercy Hospital, May 27th, 28th, and June 7th

**Published:** 1868-08

**Authors:** W. A. Barstow


### MEDICAL WARDS OF MERCY HOSPITAL, MAY 27TH, 28TH, AND JUNE 7TH.—CLINICS BY PROF. N. S. DAVIS.—TUBERCULOSIS, DELIRIUM TREMENS, ETC.—REPORTED BY W. A. BARSTOW.

The clinic hours on the 27th and 28th of May, were occupied in the examination of cases of pulmonary tuberculosis. After each member of the class had carefully examined with the stethoscope the cases presenting different stages of this destructive malady, and the physical signs had been considered, in connection with the symptoms and history of each case, the special diagnostic features of each were presented in such a manner as to indicate not only the *nature* of the disease, but its extent and stage of progress. We pass over these details, as less profitable to the readers of the EXAMINER, than to the class of students in the hospital wards.

In reference to the treatment of tuberculous cases, it was remarked, that special attention should be given to the stage of the disease and to the circumstances of each case. For, while there are a few leading indications common to all, there are secondary or special indications in each case, arising from the stage of the disease and the sanitary surroundings of the patient. To allay the morbid sensibility of the pulmonary tissue and to sustain the functions of digestion and nutrition, are indications common to all cases and in all stages of pulmonary tuberculosis. In addition to these, in the first stage it is of great importance to maintain the full capacity of the lungs for air, and to supply the blood with whatever constituents the circumstances of the patient may have rendered deficient. In the second stage, in addition to the general indications, constant vigilance is required to ward off the frequent supervention of pneumonic, bronchial, and pleuritic attacks of inflammation, which often result in the establishment of wasting hectic. In the third or suppurative stage, we have still the two leading indications mentioned, with the addition of such means as will lessen the suppurative process and prevent exhausting discharges, whether from the skin or bowels.

To sustain the functions of digestion and nutrition, regular disciplined muscular exercise in the open air, so directed as to give full expansion to the chest, is of primary and essential importance; and should be persisted in so long as the patient's strength and the absence of positive inflammation will permit. There can be no reliable substitute for this. The sleeping-room should also be of good size, dry, and well ventilated. The diet should be abundant, digestible, and nutritious, but free from mere nervous excitants or exhilarants. In the formative stage of the disease, these means alone, judiciously and perseveringly used, will often arrest its progress, and restore the patient. But if more or less cough and morbid sensitiveness of the pulmonary organs are present, it will be better to direct, in addition, the use of one of the following formulæ:

℞. Comp. Syrup of Hypophosphites,------- ℥iv.
Sulph. Morph.,----------------------- 2 grs.

Mix. Take a teaspoonful before each meal; or,

℞. Sub-Nit. Bismuth,--------------------- ʒiij.

Sub-Carb. Ferri,---------------------- ʒij.

Sulph. Morph.,------------------------ 3 grs.

Mix, divide into 30 powders, and take one before breakfast, dinner, and at bedtime.

If there be much diminution in the capacity of the lungs for air, as indicated by shortness of the inspiratory act, a tablespoonful of the following solution may be taken after each meal with much advantage, *viz.:*

℞. Chlorate Potassa,------------------------ ʒij.

Pulv. G. Arabic,------------------------- ʒij.

Aqua, ------------------------------------ ℥vj.

Mix.

When the second stage comes, characterized, as it usually is, by pains in the chest, greater severity of cough, feverishness, and other symptoms of an inflammatory nature, the following expectorant and alterative solution has been found more beneficial than any other:

℞. Muriat. Ammoniæ,------------------------ ʒiij.
Tart. Ant. et Pot.,---------------------- 2 grs.
Sulph. Morph.,--------------------------- 3 grs.
Syrup Glycyrrhiza,----------------------- ℥iv.

Mix. Take a teaspoonful every four or six hours, according to the severity of the cough and soreness.

When the third or suppurative stage is fully established, and the patient expectorates purulent or muco-purulent matter freely with commencing sweats at night, all sedatives or nauseating expectorants should be discontinued, and such tonics as promote digestion and lessen the suppurative process should be given in their place. The powder of bismuth, iron, and morphine, already given, constitutes one of the best combinations that can be used at this stage. There are now in the Hospital wards three cases in which the cough, night sweats, and expectoration have been almost stopped by these powders, while the appetite and strength have decidedly improved. Another combination, which the lecturer has long used in this stage of phthisis, consists of:

℞. Glycerine,------------------------------- ℥iij.

Syrup Iodide Iron,----------------------- ʒj.

Sulph. Morph.,--------------------------- 2 grs.

Mix. Give a teaspoonful three or four times a day.

Occasionally, cases are met with in the advanced stage, in which the tubercular disease is complicated by chronic bronchitis of such grade that it greatly increases the dyspnœa and the quantity of expectoration. A strongly-marked case of this kind was presented to the class, in Ward No. 9, not long since. In such cases, the following formula has often afforded great relief to the patient:

℞. Bal. Copiaba,------------------------------ ʒiij.

Chloroform,------------------------------ ʒiij.

Syrup Tolu,------------------------------ ʒiv.

Pulv. G. Arabic,------------------------

White Sugar,------------------------ āā ʒiij.

Rub together, and add,

Camph. Tinct. Opii,-------------------- ℥ij.

Mint Water,------------------------------ ℥ij.

Mix. Take a teaspoonful before each meal and at bedtime.

It was remarked that, in the treatment of phthisis, while the constant aim should be to give the patient plenty of pure, dry air, judicious muscular exercise, and nutritious food, much good judgment is required in selecting such remedies, from time to time, as are adapted to the various stages and complications of each case. Cod-liver oil will be found useful in all stages of the disease, provided the patient can take it without nausea or unpleasant effects upon the stomach. But the fashionable routine of treating all cases of phthisis with cod-liver oil, Bourbon whiskey, and all the rich food they can stuff down, cannot be too strongly condemned.

*Delirium Tremens.*—At the clinic of June 17th, a patient, laboring under this disease, was presented to the class; and, after calling attention to the characteristic symptoms, and the history of the case, the lecturer stated, briefly, his views of the nature and treatment of the disease. He said it was a popular belief, sustained, in part at least, by medical opinion, that the immediate cause of delirium tremens was the withdrawal of an accustomed stimulant, generally of the alcoholic class. But his own observation, extending over 30 years, had certainly shown that two-thirds of all the cases he had seen came on while the patients were still directly under the influence of these drinks, and, hence, could not be attributed to their sudden withdrawal. He believed the essential pathological condition to consist of defective nutrition, with morbid excitability of the brain-substance.

The close observer will find that so long as a drinking man or woman continues to eat regularly and digest the food taken, no symptoms of delirium occur. But when the drinking destroys the appetite, and the patient takes no food, or, if he takes it, rejects it by vomiting, in from one to three weeks his blood and tissues reach that actual impoverishment of the nutritive atoms, that, coupled with the direct irritative effects of the alcohol, tremulousness, morbid vigilance, and delirium supervene, to a greater or less extent, whether the alcoholic drink be continued or omitted. In the case here before us, the patient had not eaten a meal for 15 days, but had continued to drink liquor up to the hour of his admission into the Hospital in a state of excited delirium.

Inasmuch as the alcohol undergoes no digestion or assimilation in the human system, but circulates through it as a foreign substance, and is ultimately expelled through the organs of excretion, it adds nothing to the nutrition of the tissues. Its presence retards atomic changes, and, thereby, retains effete material, especially of the carbonaceous class, which ought to have been eliminated. Hence, under its influence, if the ingestion of food is suspended, the blood and tissues not only become impoverished of nutritive atoms, but they become surcharged with an excess of retained effete material which adds to the general functional derangements.

*Treatment.*—In the management of cases of delirium tremens, there are two well-defined objects to be accomplished, namely: to allay the morbid excitability of the nerve-tissues, so far as to procure rest; and to restore digestion and nutrition. Individual cases will present minor additional indications, from gastric and other complications, which the practitioner must meet as they arise. In the great majority of cases, the best remedy to allay the morbid excitability and mental hallucinations, so far as to induce sleep, is the bromide of potassa or the bromide of ammonium, given in from 10 grs. to 20 grs. at a dose, and repeated every two hours until the required rest is obtained. In bad cases, from $\frac{1}{3}$ to $\frac{1}{2}$ gr. of sulphate of morphia may be given each evening for the first two or three days of the treatment. The patient before the class has taken 15 grs. of the bromide of potassa every two hours during the first 36 hours, and the same dose every three and four hours from that to the present time (three days). Half a grain of morphia was given each day for the first two days.

Since the first night after the patient entered the Hospital, the delirium has been fully controlled, and convalescence is now established. Nourishment has been carefully given; first, bland and liquid, in small quantities, such as milk and meat-broths, in doses of one to three tablespoonfuls every hour, except while asleep. Subsequently, any plain food may be given at ordinary mealtimes. For many years, the lecturer has used no alcoholic drinks in the treatment of delirium tremens.

